# The Role of SNP Interactions when Determining Independence of Novel Signals in Genetic Association Studies—An Application to *ARG1* and Bronchodilator Response

**DOI:** 10.3390/jpm11020145

**Published:** 2021-02-19

**Authors:** Ryan Walsh, Kirsten Voorhies, Merry-Lynn McDonald, Michael McGeachie, Joanne E. Sordillo, Christoph Lange, Ann Chen Wu, Sharon M. Lutz

**Affiliations:** 1 Department of Population Medicine, Harvard Medical School and Harvard Pilgrim Health Care Institute, Boston, MA 02215, USA; Ryan_Walsh@harvardpilgrim.org (R.W.); Kirsten_Voorhies@harvardpilgrim.org (K.V.); rejoa@channing.harvard.edu (J.E.S.); Ann.Wu@childrens.harvard.edu (A.C.W.); 2 Division of Pulmonary, Allergy and Critical Care Medicine, Department of Medicine, University of Alabama at Birmingham, Birmingham, AL 35294, USA; mldonnelly@uabmc.edu; 3 Channing Division of Network Medicine, Brigham and Women’s Hospital and Harvard Medical School, Boston, MA 02115, USA; remmg@channing.harvard.edu; 4 Department of Biostatistics, Harvard T.H. Chan School of Public Health, Boston, MA 02115, USA; clange@hsph.harvard.edu

**Keywords:** epistasis, SNP by SNP interactions, independence, GWAS, *ARG1*, bronchodilator response

## Abstract

Genome-wide association studies (GWAS) play a critical role in identifying many loci for common diseases and traits. There has been a rapid increase in the number of GWAS over the past decade. As additional GWAS are being conducted, it is unclear whether a novel signal associated with the trait of interest is independent of single nucleotide polymorphisms (SNPs) in the same region that has been previously associated with the trait of interest. The general approach to determining whether the novel association is independent of previous signals is to examine the association of the novel SNP with the trait of interest conditional on the previously identified SNP and/or calculate linkage disequilibrium (LD) between the two SNPs. However, the role of epistasis and SNP by SNP interactions are rarely considered. Through simulation studies, we examined the role of SNP by SNP interactions when determining the independence of two genetic association signals. We have created an R package on Github called gxgRC to generate these simulation studies based on user input. In genetic association studies of asthma, we considered the role of SNP by SNP interactions when determining independence of signals for SNPs in the *ARG1* gene and bronchodilator response.

## 1. Introduction

Genome-wide association studies (GWAS) play a critical role in identifying many loci for common diseases [1] as well as complex traits [2]. Over the past decade, the rapid increase in the number of GWAS provides an extraordinary opportunity to examine the potential impact of common and rare genetic variants on complex diseases [3]. Two independent GWAS may identify two different single nucleotide polymorphisms (SNPs) in the same gene or region that are both significantly associated with the trait of interest [4]. When determining whether these two signals are independent in GWAS, epistasis and SNP by SNP interactions are often not considered.

Epistasis is defined as the interaction between different genes or SNPs and refers to the departure from independence of the effect of different genetic loci on the disease or trait of interest [5,6]. There is a complex relationship with epistasis and linkage disequilibrium (LD) [7,8]. Multiple unobserved functional polymorphisms can lead to genotyped SNPs that do not properly represent the causal variants [9] and high order LD can lead to spurious statistical epistatic associations [10]. Strong LD may suggest that detected epistasis between pairs of SNPs in different association studies need to be interpreted with caution [9,10].

Given epistasis, it is important to consider SNP by SNP interactions when determining whether genetic signals in GWAS are considered independent. For example, if an SNP is associated with the trait of interest in a GWAS, an investigator may want to determine if this SNP is an independent signal or purely the result of a correlated SNP that was previously associated with the trait of interest. In order to determine if the two signals are independent, the most popular approaches are to fit a regression of the trait of interest with the novel SNP conditional on the previously identified SNP and/or calculate LD between the two SNPs.

Through simulation studies, we examined the impact of SNP by SNP interactions when determining whether two signals are independent in a GWAS. We have created the R package, called gxgRC, which implements these simulation studies for user specified parameters. In addition, we considered the role of SNP by SNP interactions when determining independence of signals for SNPs in the *ARG1* gene and bronchodilator response in genetic association studies of asthma.

## 2. Materials and Methods

In the following simulation scenarios, we examined the impact of SNP by SNP interactions when determining the independence of two SNPs by regressing the trait of interest Y with the SNP *X*_1_ conditional on the SNP *X*_2_. We generated 1000 subjects for 5000 simulations using a significance level of 5*10^−8^. SNP *X*_1_ is generated from a binomial distribution with a binary genetic coding (i.e., dominant or recessive model) and *P*(*X*_1_ = 1) = 0.5. SNP *X*_2_ is generated from a logistic regression based on *X*_1_ such that:

(1) $$logit\left(P\left(X_{2}=1\right)\right)=\gamma_{0}+\gamma_{1}X_{1}$$

where $\gamma_{0}=0$ and $\gamma_{1}=0.3$. While *X*_1_ and *X*_2_ assume a binary genetic coding for simplicity, the results are generalizable to an additive genetic coding (i.e., *X*_j_ = 0, 1, 2 for j = 1, 2). The continuous outcome *Y* is generated from a normal distribution with a variance of 1 and a mean such that

(2) $$E\left[Y\right]=\beta_{0}+\beta_{1}X_{1}+\beta_{2}X_{2}+\beta_{I}X_{1}X_{2}$$

where $\beta_{0}=\;$0, $\beta_{1}=0.3$ or 0, $\beta_{2}=0.3$ or 0, and *β_I_* varies from 0.3 to 1 by 0.05. We considered additional simulation scenarios for different values for $\gamma_{0}$, $\gamma_{1}$ in Equation (1) and β_0_, $\beta_{1}$, $\beta_{2}$ in Equation (2). However, we observed similar results to the presented simulations scenarios; therefore, the results are not shown here. 

After the data were simulated using Equations (1) and (2), we then fit 3 algorithms and tested the following null hypotheses for each algorithm:

**Algorithm 0:** Fitting $E\left[Y\right]=\delta_{0}+\delta_{1}X_{1}$, we tested $H_{0}:\delta_{1}=0$ to determine if the SNP $X_{1}$ is associated with the trait of interest Y.

**Algorithm 1:** Fitting $E\left[Y\right]=\alpha_{0}+\alpha_{1}X_{1}+\alpha_{2}X_{2},\;\mathrm{we}\;\mathrm{tested}\;H_{0}:\alpha_{1}=0$ to determine if the SNP $X_{1}$ is associated with the trait of interest Y conditional on the SNP $X_{2}$.

**Algorithm 2:** Fitting $E\left[Y\right]=\varphi_{0}+\varphi_{1}X_{1}+\varphi_{2}X_{2}+\varphi_{I}X_{1}X_{2}$, we tested $H_{0}:\varphi_{I}=0$ to determine if there is an interaction of the 2 SNPs on the trait of interest Y.

Then, we determined when the following scenarios were true.

**Scenario 1:** Rejected Algorithm 0 $H_{0}:\delta_{1}=0$, Algorithm 1 $H_{0}:\alpha_{1}=0$, and Algorithm 2 $H_{0}:\varphi_{I}=0$.

**Scenario 2:** Rejected Algorithm 0 $H_{0}:\delta_{1}=0$ and Algorithm 1 $H_{0}:\alpha_{1}=0$, but failed to reject $H_{0}$ for Algorithm 2.

**Scenario 3:** Rejected Algorithm 0 $H_{0}:\delta_{1}=0$ and Algorithm 2 $H_{0}:\varphi_{I}=0$, but failed to reject $H_{0}$ for Algorithm 1.

**Scenario 4:** Rejected Algorithm 0 $H_{0}:\delta_{1}=0$, but failed to reject *H*_0_ for Algorithms 1 and 2.

**Scenario 5:** Failed to reject Algorithm 0 $H_{0}:\delta_{1}=0$

## 3. Results

**Figure 1:**
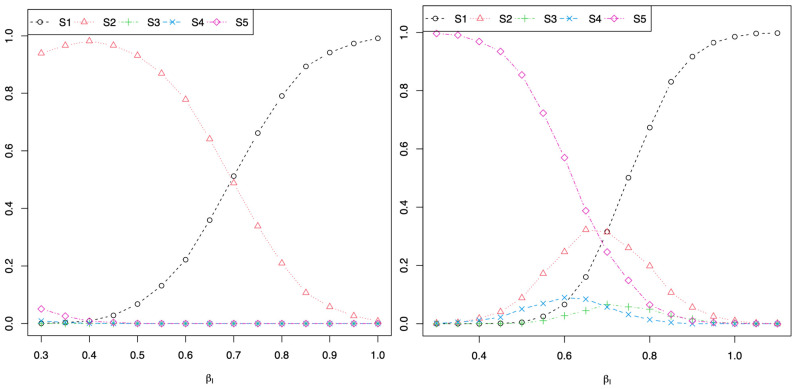
$\beta_{1}=0.3$ and $\beta_{2}=0.3$ in Equation (2) for the plot on the left and $\beta_{1}=0$ and $\beta_{2}=0$ in Equation (2) for the plot on the right, where both y-axes are the proportion of simulations where the null hypothesis was rejected. For the plot on the left, when the interaction was simulated to be weaker (i.e., *β_I_* closer to 0.3 in Equation (2)), the majority of simulations concluded scenario 2: rejecting Algorithm 0 $H_{0}:\delta_{1}=0$ and Algorithm 1 $H_{0}:\alpha_{1}=0$, but failing to reject $H_{0}$ for Algorithm 2 (i.e., there was not a signification SNP by SNP interaction). For the plot on the right, when the interaction was simulated to be weaker (i.e., *β_I_* closer to 0.3 in Equation (2)), the majority of simulations concluded scenario 5: failing to reject Algorithm 0 $H_{0}:\delta_{1}=0$ (i.e., the SNP $X_{1}$ was not associated with the trait of interest Y). For both plots, when a stronger interaction between the 2 SNPs was simulated (i.e., *β_I_* closer to 1 in Equation (2)), the majority of simulations concluded scenario 1: rejecting Algorithm 0 $H_{0}:\delta_{1}=0$, Algorithm 1 $H_{0}:\alpha_{1}=0$, and Algorithm 2 $H_{0}:\varphi_{I}=0$.

In Figure 1, $\beta_{1}=0.3$ and $\beta_{2}=0.3$ in Equation (2) for the plot on the left and $\beta_{1}=0$ and $\beta_{2}=0$ in Equation (2) for the plot on the right. For both plots and simulations, when a stronger interaction between the two SNPs is generated (i.e., *β_I_* closer to 1 in Equation (2)), the majority of simulations concluded scenario 1: rejecting Algorithm 0 $H_{0}:\delta_{1}=0$, Algorithm 1 $H_{0}:\alpha_{1}=0$, and Algorithm 2 $H_{0}:\varphi_{I}=0$. These simulations show that there can be a significant association between the SNP $X_{1}$ and the trait of interest Y in Algorithm 0, and the SNP $X_{1}$ is still significantly associated with the trait of interest Y when conditioning on the SNP $X_{2}$ in Algorithm 1. However, there is a significant interaction between the two SNPs in Algorithm 2. This shows that if a researcher were to use Algorithm 1 to conclude that the two SNPs are independent since the SNP $X_{1}$ is significantly associated with the trait of interest Y conditional on the SNP $X_{2}$, a false conclusion would be reached because there is a significant interaction of the two SNPs on the trait Y in Algorithm 2 and as generated by the data using Equation (2), such that $\beta_{I}\neq 0$. These simulations demonstrate that it is not sufficient to consider independence of two genetic signals by considering Algorithm 1: $E\left[Y\right]=\alpha_{0}+\alpha_{1}X_{1}+\alpha_{2}X_{2}\;\mathrm{and}\;\mathrm{testing}\;H_{0}:\alpha_{1}=0$. One needs to also consider if there is a significant SNP by SNP interaction by fitting Algorithm 2: $E\left[Y\right]=\varphi_{0}+\varphi_{1}X_{1}+\varphi_{2}X_{2}+\varphi_{I}X_{1}X_{2}$ and testing $H_{0}:\varphi_{I}=0$.

## 4. Data Analysis

To illustrate the effect of SNP by SNP interactions when determining conditional independence of genetic signals, we considered SNPs in chromosome 6 [*ARG1*], which has previously been associated with bronchodilator response (BDR) in asthma [11]. In the CAMP (*N* = 560) [12], CARE (*N* = 206) [13,14], and LODO (*N* = 126) [15] cohorts, we used weighted least squares regression to examine the conditional effect of four SNPs in the *ARG1* gene on BDR among subjects of European ancestry (total *N* = 892) adjusting for cohort, age, sex, body mass index (BMI) as a categorical variable (obese, overweight, vs. normal/underweight), and genetic ancestry. We picked these covariates based on other studies of BDR [16,17,18], as BDR has been found to differ depending on age [19,20], sex [21] and BMI [22]. Three of these SNPs are common variants that have previously been associated with BDR (rs2781659, rs2781663, rs2781665) [11] and one SNP (rs185631674) is a rare variant in the region.

Based on previous studies [11], rs2781659 had the most significant association with BDR in *AGR1*. In our cohorts, rs2781659 was associated with BDR adjusting for cohort, age, sex, BMI as a categorical variable (obese, overweight, vs. normal/underweight), and genetic ancestry (minor allele frequency (MAF) = 0.32, Beta = −0.15, sd = 0.05, *p*-value = 0.0037). In order to determine if the association of rs2781659 with BDR is independent of the other three SNPs, we considered the following algorithms:

**Algorithm 1:** $E\left[Y\right]=\alpha_{0}+\alpha_{1}X_{1}+\alpha_{2}X_{2}+\alpha_{C}C^{T}\;\mathrm{and}\;H_{0}:\alpha_{1}=0$

**Algorithm 2:**$E\left[Y\right]=\varphi_{0}+\varphi_{1}X_{1}+\varphi_{2}X_{2}+\varphi_{I}X_{1}X_{2}+\varphi_{C}C^{T}$ and $H_{0}:\varphi_{I}=0,$

where *C* is a vector of the covariates: cohort, age, sex, BMI as a categorical variable (obese, overweight, vs. normal/underweight), and genetic ancestry.

As seen in Table 1, the association between rs2781659 and BDR was still significant when conditioning on rs2781663 (*p* = 0.003) and rs185631674 (*p* = 0.004) but not when conditioning on rs2781665 (*p* = 0.78). The SNP by SNP interaction on BDR was marginally significant for rs2781663 (*p* = 0.06) and rs2781665 (*p* = 0.06), and significant for rs185631674 (*p* = 0.03). However, rs2781659 is in LD with rs2781663 (*r^2^* = 0.995) and rs2781665 (*r^2^* = 0.891) and rs185631674 is a rare variant in a study with a small sample size (*N* = 892).

This shows that if a researcher were to only consider Algorithm 1, where the association of rs2781659 with BDR is still significant conditioning on rs2781663 and rs185631674, the researcher would falsely conclude that there is more than one independent genetic signal with BDR. This false conclusion would not be reached with the SNP by SNP interaction considered in Algorithm 2 as well as the LD as measured by *r*^2^. This also shows that special consideration needs to be given to rare variants.

## 5. Discussion

Through simulation studies and a data analysis of SNPs in *ARG1* with BDR, we demonstrate that it is not sufficient to consider independence of two genetic signals by considering Algorithm 1: $E\left[Y\right]=\alpha_{0}+\alpha_{1}X_{1}+\alpha_{2}X_{2}\;\mathrm{and}\;\mathrm{testing}\;H_{0}:\alpha_{1}=0$. One needs to also consider whether there is a significant SNP by SNP interaction by fitting Algorithm 2: $E\left[Y\right]=\varphi_{0}+\varphi_{1}X_{1}+\varphi_{2}X_{2}+\varphi_{I}X_{1}X_{2}$ and testing $H_{0}:\varphi_{I}=0$ and/or calculating an estimate of LD, such as *r*^2^ or D’. Also, prior knowledge, for example protein–protein interactions or biological pathways, should be considered when examining SNP by SNP interactions [23].

There are some potential limitations for our simulation studies and data analysis. For the data analysis, all subjects are of European origin. A more diverse population could provide varying results, which should be considered for future analyses. Additionally, our data analysis considered genetic association studies of asthma but there is opportunity to explore the role of SNP by SNP interactions in conditional analyses by examining other diseases or traits. The sample size for the data analysis was relatively moderate (*n* = 892). Since the power to detect an interaction is substantially smaller than detecting a main effect, it should be noted that a larger sample size may be needed or the study may be underpowered to detect SNP by SNP interactions. While we have only presented two simulation studies here, we have created an R package on Github called gxgRC (https://github.com/SharonLutz/gxgRC (accessed on 12 December 2020)) [24] to generate similar simulations based on user input.

Understanding the role of epistasis and SNP by SNP interactions is important for the development of pharmacogenetic tests and personalized medicine. To date, studies in asthma pharmacogenetics have not resulted in clinical practice changes; however, exploring the role of SNP by SNP interactions has the potential to increase the likelihood of translatable findings.

## Figures and Tables

**Table 1:** Algorithm 1 considers the association of rs2781659 with bronchodilator response (BDR) conditioning on the SNPs in column 1 and Algorithm 2 considers the interaction of rs2781659 with the SNPs in column 1 on BDR. MAF denotes the minor allele frequency and SD denotes the standard deviation in the table below.

				Algorithm 1			Algorithm 2		
SNP	Position (Hg38)	*r^2^*	MAF	Beta	SD	*p*-Value	Beta	SD	*p*-Value
rs2781663	131571207	0.995	0.32	−0.15	0.05	0.003	0.14	0.08	0.06
rs2781665	131572107	0.891	0.31	−0.18	0.63	0.78	0.14	0.08	0.06
rs185631674	131570984	0.002	0.001	−0.15	0.05	0.004	−1.75	0.83	0.03

## Data Availability

The data presented in this study (CAMP, CARE, and LODO cohorts) are openly available in dbGaP at https://www.ncbi.nlm.nih.gov/projects/gap/cgi-bin/study.cgi?study_id=phs000166.v2.p1.
